# 4-(4-Nitro­benzene­sulfonamido)pyridinium nitrate

**DOI:** 10.1107/S160053680803883X

**Published:** 2008-11-22

**Authors:** Liang-Bin Hu, Yu-Xiang Ma, Chang-Zhong Liu, Ji-Guo Yang

**Affiliations:** a School of Food Science, Henan Institute of Science and Technology, Xinxiang 453003, People’s Republic of China; b College of Grain and Food, Henan University of Technology, Zhengzhou 450052, People’s Republic of China; c School of Animal Science, Henan Institute of Science and Technology, Xinxiang 453003, People’s Republic of China; d College of Light Industry and Food Science, South China University of Technology, Guangzhou 510640, People’s Republic of China

## Abstract

A short C—N distance [1.394 (2) Å] in the title compound, C_11_H_10_N_3_O_4_S^+^·NO_3_^−^, is indicative of some conjugation of the sulfonamide π electrons with those of the pyridinium ring. The crystal structure is stabilized by N—H⋯O hydrogen bonds.

## Related literature

For zwitterionic forms of *N*-aryl­benzene­sulfonamides, see: Li *et al.* (2007 ▶); Yu & Li (2007 ▶). For reference geometrical data, see: Allen *et al.* (1987 ▶). Damiano *et al.* (2007 ▶) describe the use of pyridinium derivatives for the construction of supra­molecular architectures.
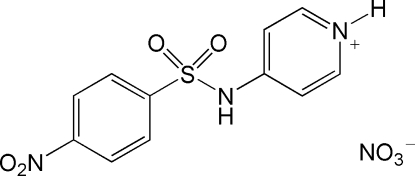


## Experimental

### 

#### Crystal data

C_11_H_10_N_3_O_4_S^+^·NO_3_^−^
*M* *_r_* = 342.29
Monoclinic, 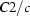

*a* = 36.516 (7) Å
*b* = 5.3742 (11) Å
*c* = 13.964 (3) Å
β = 99.54 (3)°
*V* = 2702.5 (10) Å^3^
*Z* = 8
Mo *K*α radiation
μ = 0.29 mm^−1^
*T* = 113 (2) K
0.20 × 0.12 × 0.04 mm

#### Data collection

Rigaku Saturn CCD area-detector diffractometer
Absorption correction: multi-scan (*CrystalClear*; Rigaku, 2005 ▶) *T*_min_ = 0.94, *T*_max_ = 0.99
11804 measured reflections
3203 independent reflections
2648 reflections with *I* > 2σ(*I*)
*R*_int_ = 0.037

#### Refinement

*R*[*F* ^2^ > 2σ(*F* ^2^)] = 0.040
*wR*(*F* ^2^) = 0.107
*S* = 1.09
3203 reflections
216 parameters
H atoms treated by a mixture of independent and constrained refinement
Δρ_max_ = 0.30 e Å^−3^
Δρ_min_ = −0.41 e Å^−3^

### 

Data collection: *CrystalClear* (Rigaku, 2005 ▶); cell refinement: *CrystalClear*; data reduction: *CrystalClear*; program(s) used to solve structure: *SHELXS97* (Sheldrick, 2008 ▶); program(s) used to refine structure: *SHELXL97* (Sheldrick, 2008 ▶); molecular graphics: *SHELXTL* (Sheldrick, 2008 ▶); software used to prepare material for publication: *SHELXTL*.

## Supplementary Material

Crystal structure: contains datablocks global, I. DOI: 10.1107/S160053680803883X/bg2225sup1.cif

Structure factors: contains datablocks I. DOI: 10.1107/S160053680803883X/bg2225Isup2.hkl

Additional supplementary materials:  crystallographic information; 3D view; checkCIF report

## Figures and Tables

**Table 1:** Hydrogen-bond geometry (Å, °)

*D*—H⋯*A*	*D*—H	H⋯*A*	*D*⋯*A*	*D*—H⋯*A*
N2—H2⋯O5	0.79 (2)	1.92 (2)	2.7020 (19)	171 (2)
N1—H1⋯O5^i^	0.92 (2)	1.93 (2)	2.7764 (19)	151 (2)
N1—H1⋯O6^i^	0.92 (2)	2.18 (3)	2.979 (2)	144.6 (19)

Symmetry code: (i) 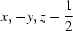.

**Fig. 1.**
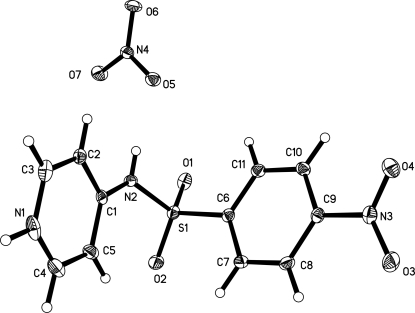
View of one molecule of the title compound showing the atom-labelling scheme. Displacement ellipsoids are drawn at the 35% probability level (arbitrary spheres for the H atoms).

## supplementary crystallographic information

### Comment

Organic pyridinium salts have been widely used in the construction of supramolecular architectures (Damiano *et al.*, 2007). As part of our ongoing studies of supramolecular chemistry involving the pyridinium rings (Li *et al.*, 2007), the structure of the title compound was determined by X-ray diffraction. In the cations of the title compound the short C—N distance [N2—C1 = 1.394 (2) Å] has a value between those of a typical C=N double and C—N single bond (1.47–1.50 Å and 1.34–1.38 Å, respectively; Allen *et al.*, 1987). This might be indicative of a slight conjugation of the sulfonamide π electrons with those of the pyridinium ring. The dihedral angle between the benzene and the pyridinium rings is 81.3 (1) °, while the the one between the nitro group and the benzene ring is 16.1 (1) °.

### Experimental

A solution of 4-nitrobenzenesulfonyl chloride (2.2 g, 10 mmol) in CH_2_Cl_2_ (10 ml) was added dropwise to a suspension of 4-aminopyridine (0.9 g, 10 mmol) in CH_2_Cl_2_ (10 ml) at room temperature with stirring. The reaction mixture was stirred overnight. The yellow solid obtained was washed with warm water to obtain the title compound in a yield of 60.5%. A colorless single-crystal suitable for X-ray analysis was obtained by slow evaporation of an nitric acid (10%) solution at room temperature over a period of a week.

### Refinement

The N-bound H atoms were located in a difference map and their coordinates were refined with *U*_iso_(H) = 1.2*U*_eq_(N). The C-bound H atoms were positioned geometrically (C—H =0.93 Å) and refined as riding with *U*_iso_(H) = 1.2*U*_eq_(C).

### Crystal data

**Table:**

C_11_H_10_N_3_O_4_S^+^·NO_3_^–^	*F*_000_ = 1408
*M**_r_* = 342.29	*D*_x_ = 1.683 Mg m^−^^3^
Monoclinic, *C*2/*c*	Mo *K*α radiation λ = 0.71073 Å
Hall symbol: -C 2yc	Cell parameters from 4207 reflections
*a* = 36.516 (7) Å	θ = 2.3–27.9º
*b* = 5.3742 (11) Å	µ = 0.29 mm^−^^1^
*c* = 13.964 (3) Å	*T* = 113 (2) K
β = 99.54 (3)º	Block, colorless
*V* = 2702.5 (10) Å^3^	0.20 × 0.12 × 0.04 mm
*Z* = 8	

### Data collection

**Table:**

Rigaku Saturn CCD area-detector diffractometer	3203 independent reflections
Radiation source: Rotating anode	2648 reflections with *I* > 2σ(*I*)
Monochromator: confocal	*R*_int_ = 0.037
Detector resolution: 7.31 pixels mm^-1^	θ_max_ = 27.9º
*T* = 113(2) K	θ_min_ = 2.3º
ω and φ scans	*h* = −47→46
Absorption correction: multi-scan(CrystalClear; Rigaku, 2005)	*k* = −7→7
*T*_min_ = 0.94, *T*_max_ = 0.99	*l* = −14→18
11804 measured reflections	

### Refinement

**Table:**

Refinement on *F*^2^	Secondary atom site location: difference Fourier map
Least-squares matrix: full	Hydrogen site location: inferred from neighbouring sites
*R*[*F*^2^ > 2σ(*F*^2^)] = 0.040	H atoms treated by a mixture of independent and constrained refinement
*wR*(*F*^2^) = 0.107	*w* = 1/[σ^2^(*F*_o_^2^) + (0.0612*P*)^2^ + 0.5371*P*] where *P* = (*F*_o_^2^ + 2*F*_c_^2^)/3
*S* = 1.10	(Δ/σ)_max_ = 0.001
3203 reflections	Δρ_max_ = 0.30 e Å^−^^3^
216 parameters	Δρ_min_ = −0.41 e Å^−^^3^
Primary atom site location: structure-invariant direct methods	Extinction correction: none

### Special details

**Table:**

Geometry. All e.s.d.'s (except the e.s.d. in the dihedral angle between two l.s. planes) are estimated using the full covariance matrix. The cell e.s.d.'s are taken into account individually in the estimation of e.s.d.'s in distances, angles and torsion angles; correlations between e.s.d.'s in cell parameters are only used when they are defined by crystal symmetry. An approximate (isotropic) treatment of cell e.s.d.'s is used for estimating e.s.d.'s involving l.s. planes.
Refinement. Refinement of *F*^2^ against ALL reflections. The weighted *R*-factor *wR* and goodness of fit *S* are based on *F*^2^, conventional *R*-factors *R* are based on *F*, with *F* set to zero for negative *F*^2^. The threshold expression of *F*^2^ > σ(*F*^2^) is used only for calculating *R*-factors(gt) *etc*. and is not relevant to the choice of reflections for refinement. *R*-factors based on *F*^2^ are statistically about twice as large as those based on *F*, and *R*- factors based on ALL data will be even larger.

### Fractional atomic coordinates and isotropic or equivalent isotropic displacement parameters (Å^2^)

**Table:**

	*x*	*y*	*z*	*U*_iso_*/*U*_eq_	
S1	0.133115 (11)	0.62515 (7)	0.45086 (3)	0.01800 (13)	
O1	0.12702 (3)	0.8195 (2)	0.51613 (8)	0.0246 (3)	
O2	0.14580 (3)	0.6801 (2)	0.36175 (8)	0.0256 (3)	
O3	0.25592 (4)	−0.2298 (3)	0.63494 (9)	0.0323 (3)	
O4	0.22026 (4)	−0.2269 (2)	0.74432 (8)	0.0285 (3)	
N1	0.06215 (5)	−0.1019 (3)	0.23971 (11)	0.0287 (4)	
N2	0.09333 (4)	0.4790 (2)	0.42774 (10)	0.0167 (3)	
N3	0.22946 (4)	−0.1490 (3)	0.66941 (10)	0.0211 (3)	
C1	0.08430 (4)	0.2815 (3)	0.36372 (10)	0.0158 (3)	
C2	0.05600 (4)	0.1211 (3)	0.38117 (11)	0.0186 (3)	
H2A	0.0441	0.1453	0.4361	0.022*	
C3	0.04565 (5)	−0.0704 (3)	0.31855 (12)	0.0259 (4)	
H3	0.0268	−0.1822	0.3304	0.031*	
C4	0.08911 (5)	0.0488 (3)	0.22111 (12)	0.0280 (4)	
H4	0.1000	0.0222	0.1648	0.034*	
C5	0.10119 (5)	0.2409 (3)	0.28211 (11)	0.0223 (4)	
H5	0.1208	0.3456	0.2694	0.027*	
C6	0.16389 (4)	0.4066 (3)	0.51584 (11)	0.0170 (3)	
C7	0.18870 (5)	0.2755 (3)	0.46944 (11)	0.0199 (3)	
H7	0.1905	0.3098	0.4037	0.024*	
C8	0.21072 (5)	0.0943 (3)	0.52069 (11)	0.0203 (3)	
H8	0.2279	0.0017	0.4909	0.024*	
C9	0.20707 (4)	0.0509 (3)	0.61656 (11)	0.0178 (3)	
C10	0.18289 (5)	0.1823 (3)	0.66407 (11)	0.0196 (3)	
H10	0.1813	0.1492	0.7301	0.023*	
C11	0.16114 (5)	0.3626 (3)	0.61267 (11)	0.0195 (3)	
H11	0.1443	0.4568	0.6432	0.023*	
O5	0.06125 (3)	0.4370 (2)	0.58801 (8)	0.0229 (3)	
O6	0.01603 (3)	0.5377 (2)	0.66295 (8)	0.0261 (3)	
O7	0.02485 (4)	0.7446 (2)	0.53506 (8)	0.0284 (3)	
N4	0.03315 (4)	0.5777 (2)	0.59463 (9)	0.0187 (3)	
H1	0.0541 (7)	−0.225 (5)	0.1953 (16)	0.050 (7)*	
H2	0.0832 (6)	0.483 (4)	0.4735 (16)	0.035 (6)*	

### Atomic displacement parameters (Å^2^)

**Table:**

	*U*^11^	*U*^22^	*U*^33^	*U*^12^	*U*^13^	*U*^23^
S1	0.0163 (2)	0.0161 (2)	0.0213 (2)	−0.00180 (15)	0.00217 (14)	0.00219 (14)
O1	0.0227 (6)	0.0169 (6)	0.0324 (6)	−0.0003 (5)	−0.0007 (5)	−0.0045 (5)
O2	0.0219 (6)	0.0286 (7)	0.0270 (6)	−0.0035 (5)	0.0062 (5)	0.0110 (5)
O3	0.0306 (7)	0.0354 (7)	0.0299 (7)	0.0162 (6)	0.0023 (5)	−0.0040 (6)
O4	0.0333 (7)	0.0257 (7)	0.0257 (6)	0.0001 (6)	0.0029 (5)	0.0056 (5)
N1	0.0367 (10)	0.0211 (8)	0.0229 (7)	0.0070 (7)	−0.0106 (6)	−0.0066 (6)
N2	0.0157 (7)	0.0185 (7)	0.0168 (6)	−0.0016 (5)	0.0049 (5)	−0.0014 (5)
N3	0.0216 (8)	0.0185 (7)	0.0216 (7)	0.0016 (6)	−0.0012 (5)	−0.0035 (5)
C1	0.0171 (7)	0.0150 (7)	0.0142 (7)	0.0036 (6)	−0.0009 (5)	0.0020 (6)
C2	0.0167 (8)	0.0189 (8)	0.0192 (7)	−0.0012 (6)	0.0000 (6)	0.0033 (6)
C3	0.0232 (9)	0.0214 (9)	0.0296 (9)	−0.0017 (7)	−0.0062 (7)	0.0016 (7)
C4	0.0365 (11)	0.0289 (9)	0.0174 (8)	0.0103 (8)	0.0013 (7)	−0.0014 (7)
C5	0.0253 (9)	0.0247 (9)	0.0174 (7)	0.0052 (7)	0.0052 (6)	0.0030 (6)
C6	0.0149 (8)	0.0173 (8)	0.0185 (7)	−0.0030 (6)	0.0015 (5)	−0.0006 (6)
C7	0.0179 (8)	0.0243 (9)	0.0183 (7)	−0.0018 (7)	0.0055 (6)	0.0005 (6)
C8	0.0169 (8)	0.0237 (9)	0.0212 (8)	0.0016 (7)	0.0058 (6)	−0.0039 (6)
C9	0.0155 (8)	0.0177 (8)	0.0193 (7)	−0.0017 (6)	0.0004 (6)	−0.0023 (6)
C10	0.0210 (8)	0.0220 (8)	0.0158 (7)	−0.0015 (7)	0.0032 (6)	−0.0023 (6)
C11	0.0192 (8)	0.0212 (9)	0.0185 (7)	0.0004 (6)	0.0048 (6)	−0.0046 (6)
O5	0.0238 (6)	0.0257 (6)	0.0203 (6)	0.0055 (5)	0.0068 (4)	0.0010 (5)
O6	0.0281 (7)	0.0321 (7)	0.0211 (6)	0.0029 (5)	0.0125 (5)	0.0057 (5)
O7	0.0311 (7)	0.0268 (7)	0.0281 (6)	0.0048 (6)	0.0070 (5)	0.0132 (5)
N4	0.0208 (7)	0.0192 (7)	0.0165 (6)	−0.0017 (6)	0.0040 (5)	0.0008 (5)

### Geometric parameters (Å, °)

**Table:**

S1—O1	1.4277 (12)	C4—C5	1.364 (2)
S1—O2	1.4286 (12)	C4—H4	0.9500
S1—N2	1.6358 (14)	C5—H5	0.9500
S1—C6	1.7687 (17)	C6—C7	1.389 (2)
O3—N3	1.2281 (18)	C6—C11	1.393 (2)
O4—N3	1.2243 (17)	C7—C8	1.385 (2)
N1—C4	1.333 (3)	C7—H7	0.9500
N1—C3	1.350 (2)	C8—C9	1.387 (2)
N1—H1	0.92 (2)	C8—H8	0.9500
N2—C1	1.392 (2)	C9—C10	1.383 (2)
N2—H2	0.79 (2)	C10—C11	1.377 (2)
N3—C9	1.472 (2)	C10—H10	0.9500
C1—C2	1.398 (2)	C11—H11	0.9500
C1—C5	1.400 (2)	O5—N4	1.2902 (17)
C2—C3	1.363 (2)	O6—N4	1.2427 (16)
C2—H2A	0.9500	O7—N4	1.2270 (17)
C3—H3	0.9500		
			
O1—S1—O2	120.81 (7)	C5—C4—H4	119.6
O1—S1—N2	104.46 (7)	C4—C5—C1	119.05 (16)
O2—S1—N2	109.44 (8)	C4—C5—H5	120.5
O1—S1—C6	108.14 (7)	C1—C5—H5	120.5
O2—S1—C6	108.30 (8)	C7—C6—C11	121.62 (15)
N2—S1—C6	104.51 (7)	C7—C6—S1	120.35 (12)
C4—N1—C3	121.75 (15)	C11—C6—S1	117.96 (12)
C4—N1—H1	118.0 (14)	C8—C7—C6	118.90 (14)
C3—N1—H1	120.2 (14)	C8—C7—H7	120.5
C1—N2—S1	126.95 (11)	C6—C7—H7	120.5
C1—N2—H2	116.5 (16)	C7—C8—C9	118.47 (15)
S1—N2—H2	110.4 (16)	C7—C8—H8	120.8
O4—N3—O3	123.99 (14)	C9—C8—H8	120.8
O4—N3—C9	118.02 (14)	C10—C9—C8	123.25 (15)
O3—N3—C9	117.99 (13)	C10—C9—N3	118.54 (14)
N2—C1—C2	117.50 (13)	C8—C9—N3	118.19 (14)
N2—C1—C5	123.52 (15)	C11—C10—C9	117.92 (14)
C2—C1—C5	118.95 (14)	C11—C10—H10	121.0
C3—C2—C1	119.22 (15)	C9—C10—H10	121.0
C3—C2—H2A	120.4	C10—C11—C6	119.82 (15)
C1—C2—H2A	120.4	C10—C11—H11	120.1
N1—C3—C2	120.27 (17)	C6—C11—H11	120.1
N1—C3—H3	119.9	O7—N4—O6	123.22 (14)
C2—C3—H3	119.9	O7—N4—O5	119.33 (13)
N1—C4—C5	120.74 (16)	O6—N4—O5	117.45 (13)
N1—C4—H4	119.6		
			
O1—S1—N2—C1	176.50 (13)	O2—S1—C6—C11	167.74 (12)
O2—S1—N2—C1	45.81 (15)	N2—S1—C6—C11	−75.67 (14)
C6—S1—N2—C1	−69.99 (14)	C11—C6—C7—C8	1.1 (2)
S1—N2—C1—C2	154.93 (12)	S1—C6—C7—C8	−175.84 (12)
S1—N2—C1—C5	−27.1 (2)	C6—C7—C8—C9	0.0 (2)
N2—C1—C2—C3	178.18 (14)	C7—C8—C9—C10	−1.0 (2)
C5—C1—C2—C3	0.1 (2)	C7—C8—C9—N3	177.55 (15)
C4—N1—C3—C2	0.9 (3)	O4—N3—C9—C10	15.6 (2)
C1—C2—C3—N1	−1.1 (2)	O3—N3—C9—C10	−165.41 (15)
C3—N1—C4—C5	0.4 (3)	O4—N3—C9—C8	−163.03 (15)
N1—C4—C5—C1	−1.4 (3)	O3—N3—C9—C8	16.0 (2)
N2—C1—C5—C4	−176.80 (15)	C8—C9—C10—C11	0.8 (2)
C2—C1—C5—C4	1.2 (2)	N3—C9—C10—C11	−177.68 (14)
O1—S1—C6—C7	−147.78 (13)	C9—C10—C11—C6	0.3 (2)
O2—S1—C6—C7	−15.24 (16)	C7—C6—C11—C10	−1.2 (2)
N2—S1—C6—C7	101.35 (14)	S1—C6—C11—C10	175.76 (13)
O1—S1—C6—C11	35.21 (14)		

### Hydrogen-bond geometry (Å, °)

**Table:**

*D*—H···*A*	*D*—H	H···*A*	*D*···*A*	*D*—H···*A*
N2—H2···O5	0.79 (2)	1.92 (2)	2.7020 (19)	171 (2)
N1—H1···O5^i^	0.92 (2)	1.93 (2)	2.7764 (19)	151 (2)
N1—H1···O6^i^	0.92 (2)	2.18 (3)	2.979 (2)	144.6 (19)

Symmetry codes: (i) *x*, −*y*, *z*−1/2.
